# Disruption of nucleocytoplasmic trafficking of cyclin D1 and topoisomerase II by sanguinarine

**DOI:** 10.1186/1471-2121-7-13

**Published:** 2006-03-02

**Authors:** Jon Holy, Genelle Lamont, Edward Perkins

**Affiliations:** 1 Department of Anatomy, Microbiology, and Pathology, University of Minnesota Medical School Duluth, 1035 University Avenue, Duluth, MN 55812-2487, USA

## Abstract

**Background:**

The quaternary isoquinoline alkaloid sanguinarine is receiving increasing attention as a potential chemotherapeutic agent in the treatment of cancer. Previous studies have shown that this DNA-binding phytochemical can arrest a number of different types of transformed cells in G0/G1, and upregulate the CKIs p21 and p27 while downregulating multiple cyclins and CDKs. To more closely examine the responses of some of these cell cycle regulatory molecules to sanguinarine, we used immunocytochemical methods to visualize cyclin D1 and topoisomerase II behavior in MCF-7 breast cancer cells.

**Results:**

5 – 10 μM sanguinarine effectively inhibits MCF-7 proliferation after a single application of drug. This growth inhibition is accompanied by a striking relocalization of cyclin D1 and topoisomerase II from the nucleus to the cytoplasm, and this effect persists for at least three days after drug addition. DNA synthesis is transiently inhibited by sanguinarine, but cells recover their ability to synthesize DNA within 24 hours. Taking advantage of the fluorescence characteristics of sanguinarine to follow its uptake and distribution suggests that these effects arise from a window of activity of a few hours immediately after drug addition, when sanguinarine is concentrated in the nucleus. These effects occur in morphologically healthy-looking cells, and thus do not simply represent part of an apoptotic response.

**Conclusion:**

It appears that sub-apoptotic concentrations of sanguinarine can suppress breast cancer cell proliferation for extended lengths of time, and that this effect results from a relatively brief period of activity when the drug is concentrated in the nucleus. Sanguinarine transiently inhibits DNA synthesis, but a novel mechanism of action appears to involve disrupting the trafficking of a number of molecules involved in cell cycle regulation and progression. The ability of sub-apoptotic concentrations of sanguinarine to inhibit cell growth may be a useful feature for potential chemotherapeutic applications; however, a narrow effective range for these effects may exist.

## Background

Investigation into the mechanisms of action of plant-derived compounds remains an important approach in the search for new and more effective anti-cancer agents. Productive sources of chemopreventative and chemotherapeutic phytochemicals include plants and plant products associated with the diet and with traditional medicinal approaches. Significant work has been conducted on species used in traditional Chinese and Ayurvidic medicine, but comparatively little attention has been paid to plants used in traditional Native American medicine. To learn more about these types of phytochemicals, we examined the effects of a number of terpenes and alkaloids present in traditional Native American medicine preparations. Ursolic and oleanolic acids, berberine, and sanguinarine were initially studied because they are prominent components in a number of plant species used in these practices. For example, a syrup called "was-a-mos," composed of the roots of spiken, sweet fern, yellow dock, elecampane, vervain, pigeon cherry, white pine bark, and bloodroot was used by the Green Bay Indians to treat cancer [1]. In addition, tribes such as the Cherokee, East Coast, and Lake Superior Indians utilized bloodroot extensively as a dye in body paint for ritual ceremonies, and in traditional medicine to treat sore throats, cough, rheumatoid arthritis and various cancers. Our preliminary results, together with other published works, indicated that sanguinarine is particularly interesting in terms of possible chemotherapeutic applications.

Sanguinarine (13-methyl benzodioxolo{5,6-c}-1,3-dioxolo{4,5-i}phenanthridinium) is an benzophenanthridine alkaloid derived from the root *of Sanguinaria canadensis *and other poppy-fumaria species, and has been shown to possess antimicrobial, anti-inflammatory, and antioxidant properties. Structurally related alkaloids are already important chemotherapeutics in the treatment of cancer, including irinotecan and topotecan [2]. Sanguinarine can block proliferation and induce apoptosis in a number of different transformed and malignant cell types [3-5]. Of particular interest from a chemotherapeutic standpoint, sanguinarine suppresses the growth of squamous carcinoma cells more effectively than normal foreskin keratinocytes [6], and inhibits the growth of a number of multidrug resistant cell lines [5].

Sanguinarine exerts multiple effects within cells, including reacting with nucleophilic and anionic groups of amino acids; binding to microtubules [7]; inhibiting certain protein kinases and phosphatases [8,9], NF-kB [10], Na+, K+-ATPase, succinate dehydrogenase and NADH dehydrogenase [11,12]; altering mitochondrial respiration and uncoupling oxidative phosphorylation [13]; forming labile covalent bonds with SH groups and inhibiting SH-containing proteins [14]; intercalating into GC-rich regions of DNA [15,16]; and inhibiting reverse transcriptase and DNA synthesis [17]. With respect to effects on the proliferative ability of malignant cells, sanguinarine has been reported to arrest the cell cycle and increase the percentage of cells in G0/G1. These effects are accompanied by an increase in cyclin-dependent protein kinase inhibitor (CKI) expression and a decrease in cyclin D1, D2 and E, and CDK2, 4 and 6 quantity [4]. The closely related alkaloid coptisine has been reported to impart a double G1 and G2/M block, and also suppresses the quantity of cyclin D1 [18]. Similar effects have been noted for certain triterpenoids, such as CDDO (2-cyano-3,12-dioxooleana-1,9-dien-28-oic acid). Differential cDNA array analyses indicate that p21^Waf1/Cip1 ^is up-regulated by CDDO treatment, and that cyclin D1 and proliferating cell nuclear antigen (PCNA) are down-regulated [19]. Both coptisine and CDDO promote proteasome-mediated destruction of cyclin D1, and threonine 286 of cyclin D1 is critical in CDDO-mediated proteolysis [18,20]. CDDO, but not coptisine, also promotes cyclin E proteolysis [18,20], suggesting that these compounds exert distinct effects on different cyclin/CDK family members.

A number of different alkaloids are potent inhibitors of topoisomerase activity. The best known examples are derivatives of camptothecin, but a number of novel isoquinoline alkaloids are receiving increasing attention, including Ecteinascidin-743, Phthalascidin, Lamellarin D, and Tetrandrine [21-25]. Interestingly, triterpenoids somewhat similar in structure to sanguinarine have also been shown to also inhibit topoisomerase activity [26-30]. These compounds exhibit cytotoxicity toward a number of different tumor cell types, including neuroblastoma [31], brain [32], skin [33] and melanoma cells [34].

Although studies to date indicate that cyclin quantity and topoisomerase activity are affected by certain phytochemicals, little is known about the detailed behavior of these cell cycle-associated molecules during exposure to these compounds. For example, it has been shown that relocalization of topoisomerases within the cell can be a critical factor in the response to topoisomerase poisons [35,36]. These observations suggest that sanguinarine and related molecules should be more closely examined for their effects on the overall behavior of cyclins, topoisomerases, and other cell cycle regulatory molecules in malignant cells. For this purpose, we treated MCF-7 breast cancer cells with sub-apoptotic doses of sanguinarine and studied cyclin D1 and topoisomerase II behavior by immunocytochemical methods. We found that sanguinarine induces a striking disruption of normal cyclin and topoisomerase II trafficking, which may represent an important part of the mechanism by which this phytochemical suppresses the proliferation of transformed cells.

## Results

**Figure 1:**
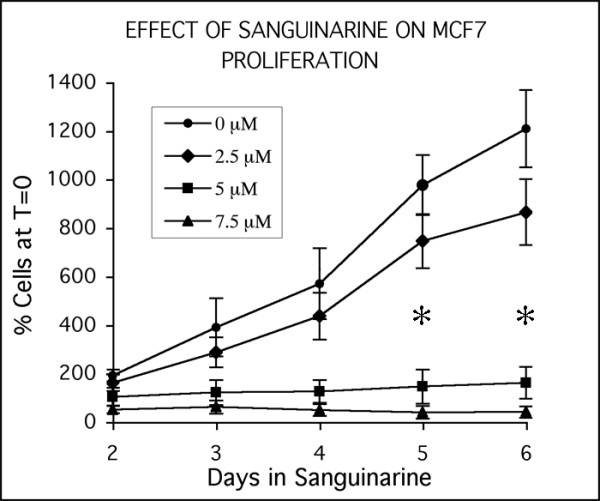
**Dose-response characteristics of MCF-7 cells treated with sanguinarine**. Responses of cells to a single dose of sanguinarine, showing effective suppression of cell growth in cultures receiving 5 or 7.5 μM sanguinarine. Data points are expressed as percentages of the cell density at time of sanguinarine addition (set at 100%, and called T = 0). Each data point is the average of three independent experiments; bars indicate standard error. Cultures treated with 5 and 7.5 μM sanguinarine for 5 and 6 days contain significantly fewer cells from control cultures and cultures treated with 2.5 μM sanguinarine (asterisks; Student's *t*-test, p < 0.05).

Dose-response studies indicate that concentrations of 2.5 μM and higher sanguinarine suppress the growth of MCF-7 cells (Fig. 1). There appears to be a relatively narrow window of growth-suppressive activity, with concentrations less than 2.5 μM exhibiting little effect, and 10 μM or higher resulting in a significant level of cell death in this cell line (data not shown). In addition, the effectiveness of sanguinarine is strongly influenced by cell density, with higher density cultures exhibiting a noticeable resistance to the same concentrations of sanguinarine effective in lower density cultures. Under the conditions used for this study, a single dose of 5 μM sanguinarine essentially completely suppressed MCF-7 proliferation for at least six days (Fig. 1), while inducing only modest levels of cell death.

**Figure 2:**
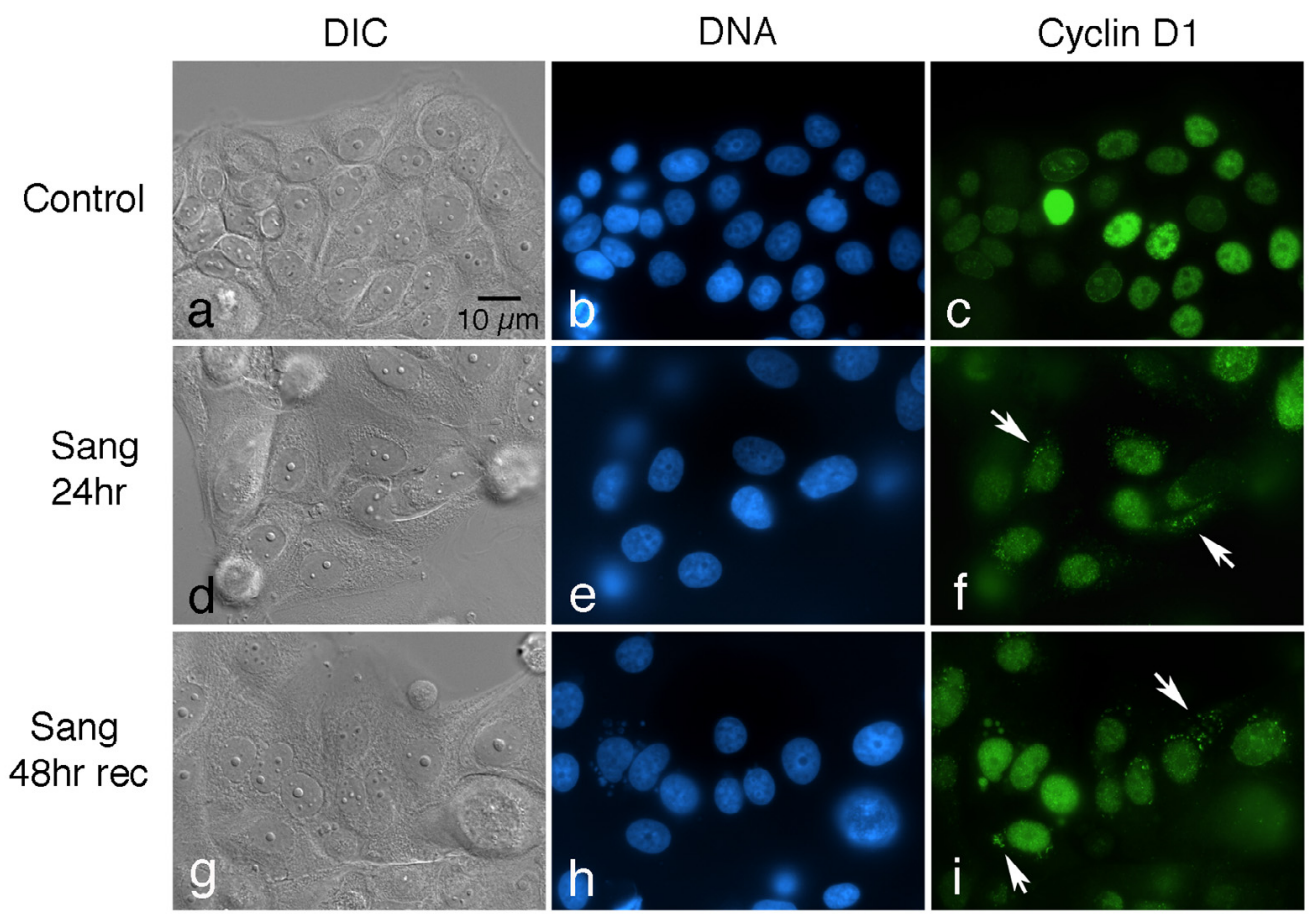
**Sanguinarine induces long-term disruptions in cyclin D1 trafficking**. Cultures were fixed and immunolabeled with an antibody to cyclin D1 (green) and counterstained with Hoechst to visualize DNA (blue). DIC, differential interference microscopy. (a-c) Control cells, showing that cyclin D1 labeling is restricted to nuclei, which vary in labeling intensity. (d-f) Culture treated with 5 μM sanguinarine for 24 hours. Note the appearance of bright cytoplasmic speckles of cyclin D1 immunofluorescence (arrows), while the overall nuclear labeling intensity appears to be diminished. (g-i) Culture allowed to recover in drug-free media for 48 hours after a 24 hour pulse of 5 μM sanguinarine. Cytoplasmic deposits of cyclin D1 (arrows) are still present in many cells.

To test for effects on certain cell cycle regulatory molecules, cultures were treated with 5 μM sanguinarine, fixed, and immunolabeled with various antibodies. Interestingly, changes in the localization patterns of cyclin D1 and topoisomerase II were noted. In control cells, cyclin D1 immunoreactivity is largely restricted to nuclei, which exhibit a range of labeling intensities (Fig. 2a–c). In cultures treated with 5 μM sanguinarine for 24 hours, however, punctate cytoplasmic deposits of immunoreactive material are evident, along with a general decrease in nuclear labeling (Fig. 2d–f). To test how persistent this cytoplasmic relocalization of cyclin D1 is, cells were treated with sanguinarine for 24 hours, followed by drug removal and recovery for 48 hours in drug-free medium. These experiments demonstrate that cytoplasmic foci of cyclin D1 are still present in many cells after two days of drug recovery (Fig. 2g–i).

**Figure 3:**
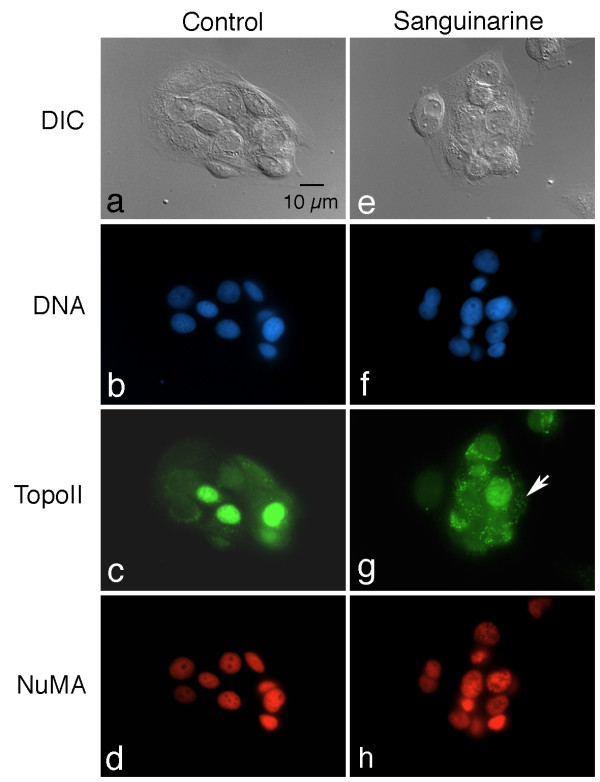
**Topoisomerase II, but not NuMA, is also aberrantly localized following sanguinarine treatment**. Cultures were fixed and double-labeled with antibodies to topoisomerase II (green), NuMA (red), and counterstained with Hoechst to visualize DNA (blue). DIC, differential interference microscopy. (a-d) Control culture, showing normal distribution of topoisomerase II and NuMA. Note variable labeling of nuclei with topoisomerase antibody; cytoplasmic labeling, when present, is homogeneous throughout the cell. In contrast, NuMA is much more uniform in its distribution throughout all nuclei. (e-h) Culture treated with 5 μM sanguinarine for 24 hours. Similar to cyclin D1, topoisomerase II is also redistributed into numerous cytoplasmic speckles (arrow). NuMA remains uniformly labeled in all nuclei.

The distribution of topoisomerase II is also altered following sanguinarine treatment (Fig. 3). In control cells, topoisomerase II immunoreactivity is weakly distributed throughout the cytoplasm, and is also present in the nucleus of many cells; some cells display very strong nuclear labeling (Fig. 3a–c). Similar to the results for cyclin D1, cytoplasmic deposits of topoisomerase II become apparent after 24 hours of treatment with 5 μM sanguinarine (Fig. 3e–g). In many cultures, this redistribution of topoisomerase II is even more pronounced than that of cyclin D1. Careful examination of double-labeled cells indicates that most, but not all, of the cyclin D1 and topoisomerase cytoplasmic immunoreactive foci appear to be co-localized.

To examine whether sanguinarine causes a non-specific redistribution of nuclear proteins in general, cultures were also labeled with antibodies to the nuclear matrix proteins lamin B1 and NuMA. Changes in the location or overall labeling pattern were not evident for either NuMA (Fig. 3d,h) or lamin B1 (not shown) in sanguinarine-treated cultures.

**Figure 4:**
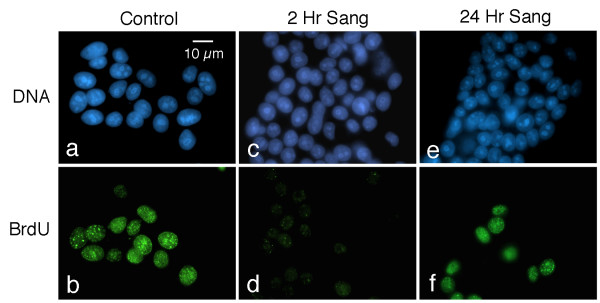
**Sanguinarine transiently inhibits DNA synthesis**. Control and drug-treated cells were pulsed with 10 μM BrdU for 30 minutes prior to fixation, and subsequently labeled with an antibody to BrdU (green) and counterstained with Hoechst (blue). (a and b) Note strong BrdU labeling of nuclei of S-phase cells. (c and d) Cells pulsed with BrdU 2 hours after the addition of 5 μM sanguinarine to the culture medium. Note the strong suppression of BrdU labeling. (e and f) Cells cultured in the presence of 5 μM sanguinarine for 24 hours prior to being pulsed with BrdU. Interestingly, a number of cells again display brightly labeled nuclei.

We then asked whether the relocalization of cyclin D1 and topoisomerase II in sanguinarine-treated cultures is accompanied by changes in the ability of these cells to carry out DNA synthesis. Following a 30 minute pulse of bromodeoxyuridine (BrdU), control cultures exhibit many strongly labeled cells, which display characteristic patterns of S-phase BrdU immunoreactivity (Fig. 4a,b). However, 2 hours of treatment with 5 μM sanguinarine markedly suppresses the ability of MCF-7 cells to incorporate BrdU into their DNA. Only a few weakly staining BrdU-positive foci remain in these cultures (Fig. 4c,d). Cultures pulsed with BrdU after 24 hours of sanguinarine treatment once again display many strongly labeled nuclei (Fig. 4e,f).

**Figure 5:**
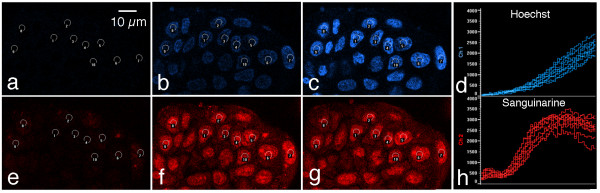
**Vital imaging of sanguinarine uptake and distribution in MCF-7 cells by laser-scanning confocal microscopy**. 5 μg/ml Hoechst and 10 μM sanguinarine were simultaneously added to living cultures and images taken with both blue (Hoechst) and red (sanguinarine) channels every 2 minutes for two hours. (a-c) Three frames from a time lapse movie (a, b and c = 6, 60, and 120 minutes after drug addition, respectively), showing Hoechst fluorescence. Using the Nikon C1 software, the average pixel intensities of circles encompassing approximately the central third of each of 10 nuclei were charted over time (d). Over this timecourse, Hoechst nuclear fluorescence progressively increases. (e-g) Three frames from the same time-lapse movie and same timepoints shown in (a-c), demonstrating that sanguinarine uptake and disposition differs from that of Hoechst. Nuclei are more rapidly labeled by sanginarine, but this signal is shorter-lived, and begins to noticeably diminish after a few hours (h).

Because sanguinarine is itself fluorescent, its uptake and distribution in living MCF-7 cells were studied with vital epifluorescence imaging methods. For these experiments, both Hoechst 33342 and sanguinarine were added to cultures to compare their uptake and distribution patterns. Both compounds could be visualized and differentiated using different lasers and filter sets provided with a Nikon C1 confocal microscope. Time-lapse studies show that cells take up Hoechst over 2 hours, resulting in a progressive increase in fluorescence that is restricted to nuclei (Fig. 5a–d). Sanguinarine also strongly labels nuclei, but displays a number of significant differences from Hoechst. It is more rapidly taken up by cells, first becoming visible in small cytoplasmic aggregates a few minutes after drug addition (not shown). These aggregates decrease in intensity at about the same time nuclear fluorescence increases, and eventually only nuclear fluorescence is evident. Sanguinarine nuclear fluorescence is shorter-lived than Hoechst, and begins to decrease in intensity after a few hours (Fig. 5e–h). Sanguinarine nuclear fluorescence is noticeably diminished six hours after drug addition, and cells are essentially non-fluorescent after 24 hours of sanguinarine treatment (data not shown).

**Figure 6:**
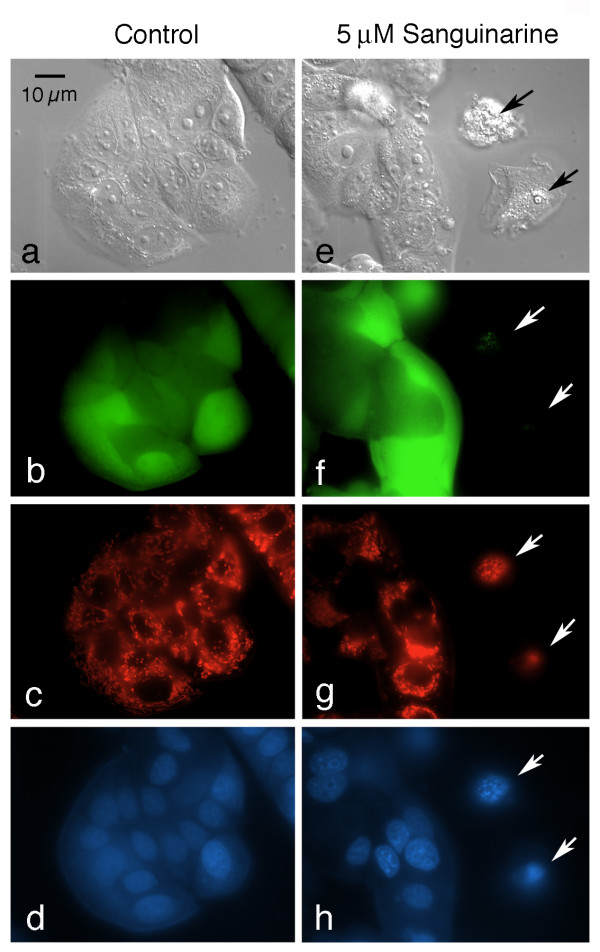
**Most cells treated with 5 μM sanguinarine for 24 hours are not apoptotic**. Multiparameter imaging was conducted with control (a-d) and sanguinarine-treated (e-h) cultures, using differential interference (DIC) microscopy (a and e), a fluorescein filter set to visualize calcein (green, b and f), a rhodamine filter set to detect both TMRM and ethidium homodimer (red, c and g), and a UV filter set to show Hoechst (blue, d and h). The same field of view is shown for each of the imaging methods for the control and drug-treated samples. Living cells fluoresce green and contain red mitochondria, but lack red nuclear labeling. In contrast, apoptotic cells do not display calcein or mitochondrial labeling, but contain nuclei with condensing chromatin that are both red (indicating ethidium homodimer penetration) and blue. Two apoptotic cells are present in the sanguinarine sample (arrows). The nucleus of the lower apoptotic cell consisted of a tightly condensed ball of heterochromatin, which is mostly out of the plane of focus in this image. Apoptotic cells can be found in both control and sanguinarine cultures, but are easily identifiable, even solely by DIC microscopy. Only healthy appearing, well-spread cells were used to show the redistribution of cyclin D1 and topoisomerase II in sanguinarine-treated cultures (Figs. 2 and 3).

To verify that most of the cells treated with 5 μM sanguinarine in the immunofluorescent studies were in fact still viable, a number of different fluorescent probes routinely used to assess the apoptotic status of cells were used, including Hoechst, tetramethylrhodamine methyl ester (TMRM), calcein AM, and ethidium homodimer. Although TMRM and ethidium homodimer both fluoresce red, they label different structures in living versus dead cells: cells with intact plasma membranes exclude ethidium homodimer and are unlabeled, whereas apoptotic cells with compromised plasma membranes exhibit red nuclei after incubation with this DNA-binding dye. In contrast, mitochondria with polarized inner membranes are strongly labeled by TMRM in living cells, whereas dying or dead cells with depolarized mitochondria are unlabeled by this probe. Calcein AM is hydrolyzed by esterases and retained in living cells with intact plasma membranes, imparting a green fluorescence, while Hoechst is a cell permeant blue fluorescent DNA-binding dye that reveals chromatin patterns in both living and dead or dying cells. Thus, living, viable cells exhibit an overall green cytoplasmic fluorescence, red mitochondia, and blue nuclei with normal patterns of euchromatin and heterochromatin, while apoptotic cells that are well along the cell death pathway lack both the green cytoplasmic calcein and the red mitochondrial TMRM signals, and contain dual-labeled red and blue nuclei that display condensed chromatin patterns. By these criteria, most MCF-7 cells treated with 5 μM sanguinarine for 24 hours (which are morphologically identical to the cells displaying altered cyclin D1 and topoisomerase II trafficking in the immunolabeling experiments), are not apoptotic (Fig. 6). Overall, a slight variation exists between groups of cells in both control and sanguinarine-treated cells with respect to both the intensity and textural labeling of heterochromatin by Hoechst. In the fields shown in Fig. 6d and 6h, the sanginarine-treated group of cells appears to be somewhat more heterochromatic and brightly stained than the controls, but the converse situation often exists, depending on the field of view. These patterns may reflect modest differences in the ability of Hoechst to penetrate individual MCF-7 cells or clones of cells at this concentration and timepoint. These subtle differences are clearly distinguishable from the extensive nuclear alterations characteristic of apoptosis.

## Discussion

Our findings indicate that a single dose of 5 μM sanguinarine results in a striking relocalization of cyclin D1 and topoisomerase II from the nucleus to the cytoplasm of MCF-7 breast cancer cells, along with an arrest of proliferation for at least six days. Previous work has largely focused on examining the effects of sanguinarine on cell viability at shorter timepoints [3-6]. A number of reports have documented the ability of sanguinarine to induce apoptosis in a number of malignant cell lines [3-5], and evidence exists that this alkaloid may exhibit some selectivity for transformed cells [6]. Although we did not specifically quantitate apoptosis in this study, a number of tests for apoptosis indicates that sanguinarine can suppress MCF-7 cell growth independently of triggering apoptosis, and that a relatively narrow window of drug activity for this effect exists, with concentrations of sanguinarine less than 2.5 μM resulting in little effect on cell viability and proliferation rate, concentrations between 2.5 and 7.5 μM suppressing cell growth without inducing significant levels of apoptosis, and higher concentrations resulting in cytotoxicity and widespread cell death. Furthermore, the window within which sanguinarine inhibits cell proliferation without inducing cell death appears to be strongly influenced by cell density, with cultures greater than about 50% confluence exhibiting more resistance to sanguinarine compared to those of lower density.

Previous reports have shown induction of apoptosis in other cell lines at lower concentrations of sanguinarine than those used in this study. For example, less than 1 μM sanguinarine is able to induce apoptosis in some cell lines [3,4]. One possible reason for this difference is that MCF-7 cells do not express functional caspase 3 due to a deletion in exon 3 [37,38], and generally show an increased resistance to many chemotherapeutic agents [39,40]. Our ongoing studies support the concept that other caspase 3-expressing cell lines (e.g., LNCaP prostate cancer cells) are indeed more sensitive to the effects of sanguinarine than are MCF-7 cells. Interestingly, in all lines so far examined, including a normal endothelial cell line immortalized by transfection with SV40 large T antigen, redistribution of cyclin D1 and topoisomerase II similar to that shown in this report has been observed at sub-apoptotic concentrations of sanguinarine (data not shown).

The relocalization of cyclin D1 and topoisomerase II from nucleus to cytoplasm demonstrates that the normal trafficking of these molecules is disrupted by sanguinarine. In our experiments, altered cyclin D1 and topoisomerase II trafficking takes a number of hours to become apparent, but is long-lasting, with cytoplasmic deposits of both molecules still evident two days after a 24 hour pulse of 5 μM sanguinarine. This does not appear to reflect a non-specific disruption of all nuclear trafficking, as demonstrated by the lack of a redistribution of lamin B1 and NuMA. Furthermore, it does not appear to simply reflect cell death, as it was observed in otherwise healthy-looking cells three days after the application of sanguinarine.

Although a large effort has been directed toward understanding the importance of cyclin expression and destruction in cell cycle regulation, it is becoming clear that regulating the trafficking and location of cyclin/CDK complexes is also a critical factor in their functioning. All of the major mitotic cyclins and their interacting CDK partners (cyclin D, A, E and B1, and CDK1,2,4 and 6) shuttle between nucleus and cytoplasm, but appear to do so by distinct mechanisms. [41-50]. The importance of proper cyclin trafficking in cell cycle control is illustrated by structural variants that display altered localization patterns. For example, splice variants of cyclin D1 have been identified that exhibit profound differences in cell cycle regulatory activity [51-53]. One such splice variant, termed D1b, is constitutively localized to the nucleus because it does not possess the necessary threonine phosphorylation modification at residue T286 required for nuclear export [45]. D1b binds and activates CDK4, and is a potent cellular transforming factor [51,53]. Site-directed mutations to the key phosphorylation site in cyclin D1 required for nuclear import, T156, act in a dominant-negative fashion, sequestering cyclin D1-CDK4 complexes in the cytoplasm [54]. Our findings that cyclin D1 is relocalized to the cytoplasm in cultures that are blocked from proliferating by 5 μM sanguinarine suggests that sub-apoptotic concentrations of this alkaloid are able to block cell growth by disrupting cyclin trafficking and reducing the overall levels of nuclear cyclin D1.

Alterations in topoisomerase II localization have been shown to critically influence the effectiveness of topoisomerase poisons [35,36]. These studies demonstrate that decreasing the nuclear:cytoplasmic ratio of topoisomerase II reduces the extent of cleavable complex formation and DNA damage imparted by topoisomerase poisons. At the same time, reduction in the quantity of nuclear topoisomerase II could impede cell division by limiting the ability to resolve the topological requirements of DNA replication. Increasing the proportion of cyclin D1 and topoisomerase II in the cytoplasm could be expected to suppress the ability of cells to enter and complete S phase, and sanguinarine has been reported to increase in the percentage of cells in G0/G1 [4].

Little information is available on the impact of sanguinarine on cell cycle traverse. Although prostate carcinoma cells were reported to accumulate in G0/G1 following sanguinarine treatment [4], no significant effects were noted for A431 epidermoid carcinoma cells [6]. The alkaloid Tetrandrine induces a block early in G1 [23,25], and the triterpenoids CDDO and ursolic acid have been shown to decrease the number of cells in S-phase in a number of cell lines, including breast cancer cells [19,55]. Our experiments show that exposure to 5 μM sanguinarine rapidly (within two hours) suppresses DNA synthesis in MCF-7 cells, but these cells recover at least some of their ability to synthesize DNA 24 hours after the addition of drug. This suggests that there may be two distinct effects of sanguinarine on DNA synthesis, namely, a rapid and direct inhibition of DNA synthesis (from which cells can recover), followed by a longer term reduction in the number of cells in S-phase, presumably due to some type of arrest or elicited checkpoint response at another point(s) in the cell cycle. We have not yet tested whether sanguinarine alters the numbers of cells in S-phase following recovery, and these two phenomenon could be reconciled if it turns out the total numbers of cells in S-phase are reduced following sanguinarine treatment. Sanguinarine binds to G-C rich regions of DNA [15], and can form DNA adducts [56]. Nick translation assays show that sanguinarine inhibits DNA replication by polymerase I, apparently through the formation of alkaloid-DNA complexes [17]. These interactions could underlie the rapid inhibition of BrdU incorporation into DNA observed in this study. It is also possible that the BrdU incorporation observed at the 24 hour timepoint reflects DNA repair. However, other studies visualizing DNA repair by BrdU incorporation methods show relatively few, dispersed foci of BrdU incorporation [57], in contrast to the strong and more uniform labeling characteristic of S-phase. The labeled nuclei at the 24 hour timepoint in this study were relatively strongly and homogeneously labeled, suggestive of DNA synthesis rather than repair, but further experiments are required to resolve this question.

An experimentally useful feature of sanguinarine is the fact that it is a fluorescent compound and can be detected by epifluorescence microscopy. This approach showed that sanguinarine rapidly (within 5–10 minutes) accumulates in the nucleus of HeLa cells, and nuclei are still fluorescent 3 hours after sanguinarine is washed out of the medium [58]. Our results demonstrate that sanguinarine is also readily taken up by MCF-7 cells and concentrated in the nucleus. However, we observed a distinct reduction in fluorescence intensity after a few hours, and cells cultured overnight in sanguinarine display essentially no nuclear fluorescence, which can be regained by the addition of fresh sanguinarine (data not shown). This behavior is different from that of Hoechst 33342, which progressively labels nuclei over the same time period. The finding that the effects of sanguinarine on cyclin D1 and topoisomerase II trafficking last far longer than the nuclear fluorescence of this alkaloid can be detected suggests that the transient presence of sanguinarine may have long-lasting effects on nuclear transport mechanisms. An alternative possibility is that non-fluorescent metabolites of sanguinarine persist that retain an ability to disrupt nuclear-cytoplasmic trafficking.

## Conclusion

Our results lead us to propose the following model for the events that occur when cells are exposed to sub-apoptotic concentrations of sanguinarine. Sanguinarine is taken up by cells and concentrated in the nucleus within minutes, where it rapidly inhibits DNA synthesis. After a few hours, sanguinarine begins to be metabolized, loses its fluorescence characteristics, and its nuclear effects diminish, resulting in the ability of cells to again synthesize DNA. However, the cellular events set into play by the initial presence of sanguinarine lead to a much longer-lived disruption of cyclin D1 and certain other nuclear protein trafficking patterns, resulting in extended cell cycle arrest and inhibition of cell proliferation. In terms of development as a possible cancer chemotherapeutic, this model suggests that sanguinarine may help suppress malignant cell growth even at sub-apoptotic concentrations. It will be interesting and important to transfer these experiments to an *in vivo *setting to determine if similar responses occur in malignant cells in an intact animal.

## Methods

### Cell culture

MCF-7 cells (the kind gift of Dr. Merry Jo Oursler, Department of Orthopedics, Mayo Clinic, Rochester, MN) were grown in Dulbecco's modified Eagle's medium (GIBCO, Grand Island, NY) and 7.5% Fetal Clone III (HyClone, Logan, UT) in a 5% CO_2 _atmosphere at 37°C. Cells were passaged by trypsinization using standard methods; all experiments were conducted with cultures between 25–75% confluency, and in log-phase growth.

### Cell proliferation measurements

Sulforhodamine B assays were conducted to measure the effects of sanguinarine on the proliferation of MCF-7 cells. Cells were seeded at a concentration of 2.5 × 10^4 ^cells/ml in 24-well plates, and allowed to recover for 2 days prior to drug addition. Sanguinarine (>98% pure, Sigma Chemical Co., St. Louis, MO) was diluted to 0.5 mM in DMEM from a 50 mM stock solution in DMSO, and this diluted stock solution added to multiwell plates to a final concentration of 2.5, 5, or 7.5 μM. Control wells received diluted vehicle only, corresponding to the amount present in the 7.5 μM sanguinarine wells (i.e., 15 μl of a 1% DMSO solution). At the time of drug or vehicle only addition, the medium from one column (4 wells) was aspirated and the wells subsequently rinsed with DMEM followed by sterile distilled water. The water was aspirated, and the plate replaced in the incubator (these wells represent the cell density at the time of drugging, called T = 0). At days 2, 3, 4, 5 and 6 following sanguinarine addition, this process was repeated for another column of wells (each column consisting of control, 2.5, 5, and 7.5 μM sanguinarine-treated wells). After the last column in a dish was processed (on day 6), the entire air-dried plate was fixed with absolute methanol containing 1% acetic acid (at -10°C) for 30 minutes, the methanol decanted, and the plate again air-dried. Sulforhodamine B (0.5% in 1% acetic acid) was added to each well, and the plate incubated at 35°C for 1 hour. Plates were rinsed with 1% acetic acid, air-dried, and the bound dye eluted with 1 ml of 10 mM Tris buffer, pH 10. The absorbance was measured in a spectrophotometer at 540 nm; the amount of dye released is proportional to the number of cells present in the dish, and is a reliable indicator of cell proliferation.

### Immunofluorescence labeling methods

Cells were seeded on glass coverslips in 6-well plates at a density of 2.5 × 10^4 ^cells/ml, and allowed to recover for two days prior to drug addition. 5 μM Sanguinarine was added as described above for various lengths of time, after which the cells were fixed by plunging the coverslips into cold (-10°C) absolute methanol. In some experiments, sanguinarine was washed out after 24 hours (via 3 exchanges of feeding solution), and further cultured in drug-free medium for another 24 or 48 hours prior to fixation. Cells were prepared for immunolabeling by rehydration in PBS containing 0.05% Tween-20 (PBST), and subsequently blocked with 1% nonfat powdered milk dissolved in PBST for 1 hour at 37°C. Cells were labeled with antibodies to cyclin D1 (Santa Cruz Biotechnology Inc., Santa Cruz, CA) topoisomerase II (the kind gift of Dr. Scott Kaufmann, Mayo Clinic, Rochester, MN), or NuMA (the kind gift of M. Snyder, Yale University, New Haven, NY) for 1 hour at 37°C, washed with three changes of PBST, and incubated in the appropriate fluorescently-conjugated secondary antibodies (goat-anti-mouse- or goat-anti-rabbit-fluroescein or Texas Red, Jackson Immunoresearch, Malvern, PA), containing 5 μg/ml Hoechst 33342 (Sigma Chemical Co.) for 1 hour at 37°C. After 3 final PBST washes, coverslips were mounted in an anti-fade medium (90% glycerol, 10% 0.5 M 3- [cyclohexylamino]-1-propanesulfonic acid, buffer pH 9.0, containing 0.1% paraphenylenediamine and 0.1% 1,4-diazabicyclo- [2.2.2]octane) on glass slides, and examined by routine epifluorescence or laser-scanning confocal microscopy.

### BrdU labeling

After either 2 or 24 hours of treatment with 5 μM sanguinarine, cells were pulse-labeled with 10 μM Bromodeoxyuridine (BrdU; Sigma) for 30 minutes and then fixed in cold (-10°C) absolute methanol. For BrdU labeling, cells were rehydrated with PBST and treated with 2 N HCl for 20 minutes at room temperature. Cells were then washed five times with PBST, and incubated in PBST containing 0.5% non-fat powdered milk for 30 minutes at 37°C. Anti-BrdU antibody (G3G4, Developmental Studies Hybridoma Bank, Iowa City, IA) was added for 1 hour at 37°C, and cells washed three times in PBST and incubated in goat-anti-mouse antibody conjugated to Texas Red (Jackson Immunoresearch) containing 5 μg/ml Hoechst 33342 for 1 hour at 37°C. Coverslips were finally washed in PBST and mounted as described above.

### Vital cell imaging

Cells were seeded into 35 mm glass-bottom Petri dishes (MatTek Inc., Amherst, MA) at a density of 2.5 × 10^4^/ml, and allowed to recover for 2 days prior to use. To visualize and compare cellular uptake of sanguinarine and Hoechst dye, the normal culture medium (DMEM/7.5% FBS) was exchanged with a 1:1 mixture of DMEM and Liebovitz's L-15 medium containing 5% FBS (which maintains a neutral pH in the absence of CO_2_) and placed on the stage of a Nikon C1 confocal microscope (at room temperature). Laser excitation and PMT gain were set at values previously found to be suitable for imaging sanguinarine and Hoechst in cells incubated in these compounds for 2–4 hours, and time-lapse acquisition started (sequential scanning every 2 minutes for 2–4 hours). Sanguinarine (10 μM) and Hoechst 33342 (5 μg/ml) were then simultaneously added to the dishes from freshly diluted 100× stock solutions prepared in DMEM. Hoechst fluorescence was visualized using a Coherent VioFlame solid state laser (20 mW at 405 nm) and a 480 nm dichroic long pass, with a 450/35 nm band pass for the blue channel (PMT 1); sanguinarine was visualized using a Spectra Physics Krypton-Argon Model 163C polarized laser (40 mW at 488 nm) and a 545 nm dichroic long pass, with a 605/75 band pass for the red channel (PMT 2). To test for apoptosis, cells seeded into glass-bottom dishes as described above were treated with a a cocktail of fluorescent probes, including Hoechst 33342 (5 μg/ml), 100 nM tetramethylrhodamine methylester (TMRM; Molecular Probes, Eugene, OR), 2 μM calcein AM (Molecular Probes), and 2 μM ethidium homodimer (Molecular Probes) for 45 minutes prior to imaging on an inverted fluorescent microscope.

## Abbreviations

BrdU, bromodeoxyuridine; CDDO, 2-cyano-3,12-dioxooleana-1,9-dien-28-oic acid; CDK, cyclin dependent kinase; DMEM, Dulbecco's modified Eagle's medium; PBST, phosphate buffered saline-Tween 20; PMT, photomultiplier tube; SRB, sulforhodamine B; TMRM, tetramethylrhodamine methyl ester.

## Authors' contributions

JH assisted with the experimental design, carried out some of the immunocytochemical studies, and helped with data analysis and manuscript preparation; GL initiated the sanguinarine project, performed some of the SRB and immunocytochemical studies, and assisted with manuscript preparation; EP assisted with the experimental design, the interpretation of the results, and manuscript preparation.
